# On the Effects on Cortical Spontaneous Activity of the Symmetries of the Network of Pinwheels in Visual Area V1

**DOI:** 10.1186/s13408-015-0023-8

**Published:** 2015-05-30

**Authors:** Romain Veltz, Pascal Chossat, Olivier Faugeras

**Affiliations:** Neuromathcomp Project Team, Inria Sophia Antipolis Méditerranée, 2004 Route des Lucioles-BP 93, 06902, Sophia Antipolis, France; Laboratoire J-A Dieudonné, Université Nice Sophia Antipolis, parc Valrose, 06108, Nice Cedex, France

**Keywords:** Visual hallucinations, Invariant torus, Poincaré–Hopf

## Abstract

This paper challenges and extends earlier seminal work. We consider the problem of describing mathematically the spontaneous activity of V1 by combining several important experimental observations including (1) the organization of the visual cortex into a spatially periodic network of hypercolumns structured around pinwheels, (2) the difference between short-range and long-range intracortical connections, the first ones being rather isotropic and producing naturally doubly periodic patterns by Turing mechanisms, the second one being patchy, and (3) the fact that the Turing patterns spontaneously produced by the short-range connections and the network of pinwheels have similar periods. By analyzing the PO maps, we are able to classify all possible singular points (the pinwheels) as having symmetries described by a small subset of the wallpaper groups. We then propose a description of the spontaneous activity of V1 using a classical voltage-based neural field model that features isotropic short-range connectivities modulated by non-isotropic long-range connectivities. A key observation is that, with only short-range connections and because the problem has full translational invariance in this case, a spontaneous doubly periodic pattern generates a 2-torus in a suitable functional space which persists as a flow-invariant manifold under small perturbations, for example when turning on the long-range connections. Through a complete analysis of the symmetries of the resulting neural field equation and motivated by a numerical investigation of the bifurcations of their solutions, we conclude that the branches of solutions which are stable over an extended range of parameters are those that correspond to patterns with an hexagonal (or nearly hexagonal) symmetry. The question of which patterns persist when turning on the long-range connections is answered by (1) analyzing the remaining symmetries on the perturbed torus and (2) combining this information with the Poincaré–Hopf theorem. We have developed a numerical implementation of the theory that has allowed us to produce the predicted patterns of activities, the planforms. In particular we generalize the contoured and non-contoured planforms predicted by previous authors.

## Introduction

The primary area (V1) of the visual cortex is one of the first locations targeted by connections from the thalamus which relays (and processes) inputs from the retina. In some mammals like primates, cats or ferrets (see [1, 2, 3]), this cortical area is very precisely organized in modules, called cortical hypercolumns, which process visual attributes (like local orientation, spatial frequency) of different points in the visual field. Note that there is almost no experimental evidence of hypercolumns from histology (see [4]). Most of our knowledge derives from functional evidence, i.e. when an external stimulus is applied. In this work, we focus on the processing of the local orientation of visual stimuli which is reflected by the ability of some neurons to fire only when a drifting grating of specific (called preferred) orientation is presented in their receptive field. The distribution, on the cortical sheet, of the preferred orientation of these particular neurons (see [5, 6]) defines a *Preferred Orientation map* (hereafter called the PO map) which has a near lattice structure and which is continuous except at particular points called pinwheels.

It has been argued by several authors (Ermentrout [7], Bressloff and Cowan [8, 9]) that V1 is to some extent structured like a crystal: it can be approximated by a plane where the pinwheels are arranged in a doubly periodic lattice and the main features of cortical activity in V1 can be interpreted in this framework. This idealization naturally introduces symmetries in the problem, which makes deeper analysis accessible. As long as the pinwheels are nearly arranged on a periodic lattice we can expect that the main conclusions of our analysis will remain valid.

There is also experimental evidence (see below) that the spatial distribution of connections emanating from one neuron in V1 differs according to whether the connections are *local* (within one hypercolumn) or *long-range* (between different hypercolumns). Local connections are considered to be isotropic. In the absence of long-range connections the network (or field) of local connections would be Euclidean-invariant, that is, invariant under rigid displacements and reflections in the plane, a property that is transmitted to the model equations [7]. On the other hand, long-range connections are subject to the constraint of respecting the symmetries of the PO map, thereby reducing the full Euclidean group symmetries to a crystallographic subgroup associated with the lattice of pinwheels. Moreover, experimental observations suggest that the strength of long-range connections is significantly weaker than that of local connections, which allows for treating the long-range connections as a perturbation of the local ones.

However, as shown initially by Ermentrout and Cowan [7], spontaneous activity of V1 without long-range connections also produces naturally doubly periodic steady patterns by the Turing mechanism [10]. This fact has been at the origin of a series of papers where hallucinatory patterns (seen by patients under the influence of drugs or other stimuli of the visual cortex which are not coming from the thalamus) have been explained as Turing patterns bifurcating in V1 [8, 11]. It was assumed in these papers that the lattice of pinwheels can be itself approximated as a continuum in the plane, that is, every point in the plane is a pinwheel. Then the full system of connections, local and long-range, keeps translation invariance: any planar translation applied to a pattern yields another one, up to periodicity. Therefore a pattern is not an isolated solution but rather generates a manifold of solutions under the action of translations, which is called an *orbit* under the action of the group of translations. This orbit, thanks to the periodicity of the pattern, can be identified with a 2-torus in a suitable functional space. Moreover, as long as this torus is a *normally hyperbolic* manifold (it means here that steady-state patterns are hyperbolic along directions transversal to the orbit), it persists as a flow-invariant manifold under small perturbations.

When the lattice of pinwheels is discrete, long-range connections reduce the translation group $\mathbb {R}^{2}$ to a discrete subgroup isomorphic to $\mathbb {Z}^{2}$. Our aim in this paper is to study the effect of switching on such long-range connections on the tori of solutions of the system with local connections only. As in the above cited papers we assume that the strength of long-range connections is small compared to that of local connections. The introduction of the long-range connections destroys part of the symmetries but not all of them. The perturbed torus features these remaining symmetries. We classify possible dynamics on this invariant manifold by applying topological methods (the Poincaré–Hopf theorem) together with the symmetries. Numerical (direct) simulations of the equations then allow one to determine which phase portrait is actually observed among those which have been theoretically identified.

Our motivation for this work is to understand how introducing a discrete lattice of pinwheels would modify the states and dynamics of spontaneous activity in V1, and we expect this could have some consequences on the theory of hallucinations of Bressloff and Golubitsky et al. in [8].

The problem of the effect of long-range connections with discrete translation invariance on the Turing patterns which bifurcate when these connections are inactive has been addressed by Bressloff [12] (see also Baker and Cowan [13]) in the following context. These authors assumed that the periods of the patterns and of the lattice of pinwheels were not correlated. An analysis inspired by methods initially introduced for hydrodynamical systems allowed them to build Ginzburg–Landau equations, in order to describe the slow modulation of the bifurcating patterns under the effect of spatial periodic forcing due to the long-range connections.

There are, however, some experimental observations which suggest that in fact, due to synaptic plasticity, the periods of the pinwheel lattice and of the Turing patterns are close to each other [14, 15]. It is therefore not unrealistic to assume that these periods are in fact equal, which we do in this study. Our approach differs on another point: we do not assume the system to be close to bifurcation from homogeneous state. Therefore the validity range of our results goes well beyond bifurcation analysis, and allows a rigorous mathematical treatment in the spirit of Lauterbach and Roberts [16] about forced symmetry breaking of group orbits of equilibria with spherical symmetry.

The way in which neurons in V1 acquire orientation selectivity is still controversial as the connections from the thalamus provide a very small percentage of the inputs: 95 % of these inputs are made of recurrent connections, i.e. intracortical connections (see [17]). Despite this experimental evidence there are still two extreme attitudes to account for these facts: either we assume that the recurrent connections provide much of the input to each neuron, or that each neuron mostly follows the thalamic input, discarding the recurrent connections; see [18]. Our approach lies in between.

We use the following standard neural field representation for the neural activity *V* of the neuron located at **x** in the connected domain *Ω* of $\mathbb {R}^{2}$ representing V1:

1 $$\begin{aligned} &\frac{d}{dt}V(\mathbf {x},t) = -V(\mathbf {x},t)+\int _{\varOmega}J(\mathbf {x},\mathbf {y})\mathbf {S}\bigl(V(\mathbf {y},t)\bigr)\,d \mathbf {y}+I_{\mathrm{thalamus}}(\mathbf {x},t), \\ &\quad \mathbf {S}(x) = \frac{1}{1+e^{-\sigma x+T}}, \end{aligned}$$

where **S** is the sigmoidal function with range $(0,1)$ and $T/\sigma $ is the threshold. The slope is *σ* at $x=T/\sigma$. The domain *Ω* is usually taken as the infinite plane. This is not an unrealistic approximation in the analysis, because the number of pinwheels in V1 is rather large, typically several thousands [8]. However, in numerical computations one has to choose a bounded domain and the simplest choice is a square with periodic boundary conditions: opposite sides are identified.

The connectivity function *J* represents the intracortical connections between neurons and $I_{\mathrm{thalamus}}$ the thalamic input. Following [12], we decompose the connectivity function as follows:

2 $$ J(\mathbf {x},\mathbf {y}) = J_{\mathrm{loc}}(\mathbf {x},\mathbf {y}) + \epsilon_{\mathrm {LR}}J_{\mathrm{LR}}( \mathbf {x},\mathbf {y}), \quad\epsilon_{\mathrm{LR}}\ll1 , $$

where $J_{\mathrm{loc}}$ models the local connections and $J_{\mathrm {LR}}(\mathbf {x},\mathbf {y})$ models the long-range connections. The small factor $\epsilon_{\mathrm {LR}}$ in front of $J_{\mathrm{LR}}$ accounts for the fact mentioned above that the strength of the long-range connections is significantly weaker than that of the short-range connections [19]. From [4, 15] we can assume that the local connections are isotropic (rotation invariant), and we further assume for simplicity that they are also homogeneous (translation invariant). Hence $J_{\mathrm{loc}}$ only depends on the distance between **x** and **y**:

$$J_{\mathrm{loc}}(\mathbf {x},\mathbf {y}) = J_{\mathrm{loc}}\bigl(\Vert \mathbf {x}-\mathbf {y}\Vert \bigr). $$

$\Vert \mathbf {x}-\mathbf {y}\Vert $ is the usual Euclidean norm in $\mathbb {R}^{2}$. The form of the function $J_{\mathrm{LR}}$ is discussed in Sect. 3.1. The external (thalamic) input function $I_{\mathrm{thalamus}}$ will be assumed equal to 0 throughout the paper in order to consider spontaneous activity only.

### Remark 1

For all numerical experiments, we choose

3 $$ J_{\mathrm{loc}}(\mathbf {x})=ae^{-\frac{\Vert \mathbf {x}\Vert ^{2}}{2\sigma _{\mathrm{loc}}^{2}}}-e^{-\frac {\Vert \mathbf {x}\Vert ^{2}}{4\sigma_{\mathrm{loc}}^{2}}} . $$

Indeed, from [4, 15], we know that the local connectivity is homogeneous in the cat and in the monkey. Note that we assume that the excitatory (respectively, inhibitory) connections are modeled as Gaussian with width $\sigma _{\mathrm{loc}}$ (respectively, $2\sigma_{\mathrm{loc}}$) [15]. The constant $a=4e^{-\sigma_{\mathrm {loc}}^{2}/2}$ is tuned such that the first bifurcation of the homogeneous state has a wave-vector of norm 1; see [20] for more details and for bifurcation diagrams. $\sigma_{\mathrm{loc}}$ controls the stability of stripes vs. spots.

**Fig. 1:**
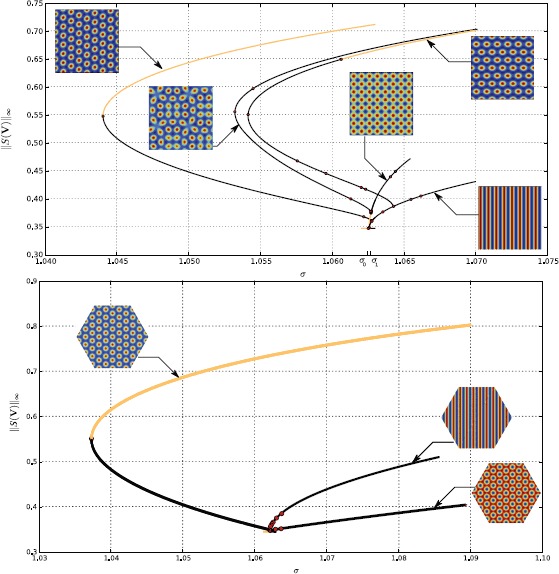
Bifurcation diagrams of (1) w.r.t. the slope parameter *σ*, when $I_{\mathrm{thalamus}}=0$ and $J(\mathbf {x},\mathbf {y})$ is a difference of Gaussians on a *square* (*above*) or *hexagonal* (*bottom*) domain with periodic boundary conditions. The *vertical axis* is the norm of the sigmoid of the stationary solution. *Black lines* correspond to unstable solutions and *brown lines* to stable ones. Each bifurcated solution yields a torus of identical steady states up to translations. *Red dots* are additional bifurcation points where the bifurcated branches have not been computed. The connectivity is tuned such that the first bifurcation point for the nonlinear gain *σ* is a supercritical $\mathbf{D}_{4}$-pitchfork (respectively, $\mathbf{D}_{6}$-pitchfork) bifurcation with wave-vector $\|\mathbf{k}\|=1$ for which the *spots* are stable, while the *stripes* are not. The size of the square cortex is $8\times 2\pi$ and the numerical mesh size is 1024^2^ (*top*) and $3\times512^{2}$ (*bottom*), the threshold is $T = 0.1$ and $\sigma_{\mathrm{loc}} = \pi\cdot0.395$. We only used the 15 eigenvalues with the largest real parts to find the bifurcation points. See Appendix B for details concerning the numerical methods

Let us have a look at the bifurcation diagram in Fig. 1, top. It shows bifurcated patterns in a square *Ω* with periodic boundary conditions when only local connections are turned on. The bifurcation parameter in abscissa is the slope *σ* of the sigmoid function **S**. It shows, as expected, two primary bifurcated branches: one of stripes and one of “spots”, which correspond to a periodic pattern with square symmetry. Near bifurcation other types of solutions branch off these primary branches. Observe that the stripes are always unstable and that the spots are stable in a small interval, up to the first secondary bifurcation. On the other hand two secondary branches of doubly periodic states bifurcate sub-critically and recover stability after bending back in the increasing *σ* direction. These states are then stable on large intervals of values of *σ*. In their stability domain they look pretty much like hexagonal patterns. In fact, they approximate the exact hexagonal patterns that would bifurcate on a domain with hexagonal symmetry (and periodic boundary conditions). If the size of *Ω* is increased, these two branches come closer to each other and bifurcate closer from the primary bifurcation point. In the limit of a domain with infinite size, they merge into one branch, which bifurcates at the same point as the spots and stripes, and they correspond exactly to the hexagonal patterns which are well known from Turing theory of pattern formation. We compare this diagram to the case where the domain *Ω* is an hexagon with periodic boundary conditions; see Fig. 1, bottom. We were able to find numerically the branches of spots (transcritical) and stripes (pitchfork). We did not compute the secondary branches because our point was to show that the hexagons (spots) are stable over an extended region.

Since we are interested in stable states, we can consider in the analysis, instead of these “imperfect hexagons”, the exact hexagonal patterns that will show up in an hexagonal lattice. This will make the analysis simpler and richer at the same time, and the results will still be relevant for the secondary “approximate” hexagonal patterns observed in Fig. 1, top. Despite the fact that square patterns (spots in Fig. 1, top) are stable only in a small region near bifurcation, we shall discuss both square and hexagonal patterns for completeness.

Our primary motivation here is to understand how the long-range connections with discrete lattice symmetry affect the dynamics of the network. This is made possible by using the fact that these connections act as a perturbation [19] of the dynamics generated by the local connections. Looking at the spontaneous activity, i.e. without thalamic input, is motivated by two reasons. The first reason is that it has been argued that the hallucinating patterns generated by some drugs can be explained by the spontaneous activity of networks [7, 8] similar to the one studied here. The second reason is that if we were to include an input with amplitude *ϵ* that we vary, it would be the same as working with a fixed amplitude but varying the slope of the sigmoid function **S** [21]. Hence, looking at the case of a thalamic input can be thought of as a deformation of the case considered here.

The paper is organized as follows. We first describe in Sect. 2 the Turing patterns that appear when the long-range connections are switched off. In Sect. 3 we compute the network symmetries that are induced by the long-range connections. We then study in Sect. 4 the perturbation of the Turing patterns, before drawing some conclusions in Sect. 5.

## Turing Patterns in the Unperturbed Case

In this section, we formulate our assumptions on the symmetries of the unperturbed solutions,^1^ written $V_{0}(\mathbf {x})$, that will be analyzed upon switching on the long-range connections. Since the computation of Turing patterns has been extensively documented in the literature (see for example [22, 23] for a review and [7] for neural fields), we will not cover it here and only briefly recall the main results. We have seen in the introduction that, when $\epsilon_{\mathrm {LR}}=0$, the system is invariant under any translation in the periodic domain *Ω*. Adjusting *Ω* to be a square or an hexagon, branches of steady patterns with square or hexagonal symmetry bifurcate from the homogeneous state as the slope *σ* of the sigmoid function **S** reaches a threshold value. These patterns are stable in some range of values of *σ* as shown in Fig. 1. Stability here means “orbital stability”. Indeed any translation applied to such a state $V_{0}$ gives another solution, thanks to the translational invariance. The group of translations acting in *Ω* (which has periodic boundaries) is the torus $\mathbb {R}^{2}/\mathbb {Z}^{2}$, which, moreover, acts faithfully on any $V_{0}$ with square or hexagonal pattern. Therefore the set (called *translation group orbit*) of translated states from $V_{0}$ is a torus manifold. Moreover, this torus is an attractor if $V_{0}$ is orbitally stable.

A central hypothesis of the current study is the assumption that the spatial period of $V_{0}$ matches the spatial period of the PO map. The experimental data that support this assumption were already mentioned in the introduction. For example, we will not study the approximate “hexagonal” patterns (in the square cortex as in Fig. 1) because they do not satisfy our assumption. Also, the stripes^2^ patterns will not be studied here. Indeed, they have less symmetries and their study would only require minor modifications of the present argument.

## Network Symmetries

**Fig. 2:**
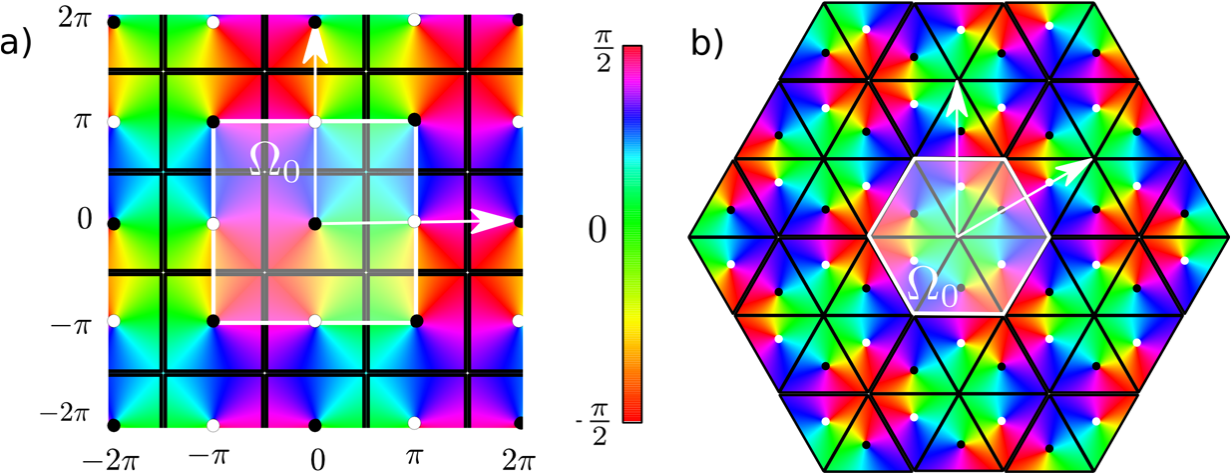
The two pinwheel lattices considered in this work in the *square* case (**a**) and in the *hexagonal* case (**b**). The *black lines* show the elementary domains (the hypercolumns), the *white lines* the fundamental domains $\varOmega_{0}$ (see text). The clockwise (respectively, counterclockwise) pinwheels are represented with *black* (respectively, *white*) *dots*. The vectors $2\pi \mathbf {e}_{1}$ and $2\pi \mathbf {e}_{2}$ are shown in *white*

For reasons which have been explained in the introduction we are interested in the perturbation of Turing patterns with square or hexagonal symmetries. Such patterns have maximal lattice symmetry, meaning that they are invariant under the dihedral group of rotations/reflections $\mathbf {D}_{k}$ with $k=4$ (squares) or 6 (hexagons), and of course they are invariant under the discrete translations which define the lattice group. It is therefore convenient to suppose that the domain *Ω* representing the primary visual cortex is a square or a hexagon with periodic boundary conditions (opposite sides are identified). Let $\mathbf {e}_{1}$, $\mathbf {e}_{2}$ be two vectors generating the lattice and $\varOmega_{0}$ be a fundamental domain centered at the origin *O* of *Ω* (see Fig. 2). We choose $\mathbf {e}_{1}$ and $\mathbf {e}_{2}$ as unit vectors, making an angle of $\pi/2$ in the square case, and of $\pi/3$ in the hexagonal case. Then

$$\varOmega_{0} = \biggl\lbrace O+x_{1}\mathbf {e}_{1} + x_{2}\mathbf {e}_{2} \Bigm| -\frac{\ell }{2}\leq x_{i} < \frac{\ell}{2} , i=1,2 \biggr\rbrace , $$

*ℓ* being an arbitrary length scale. By construction there exists an integer *N* such that, by periodicity,

$$\varOmega\simeq\lbrace\varOmega_{0}+j_{1}\mathbf {e}_{1}+j_{2} \mathbf {e}_{2} \mid j_{1},j_{2}=-N,\dots,N \rbrace. $$

**Fig. 3:**
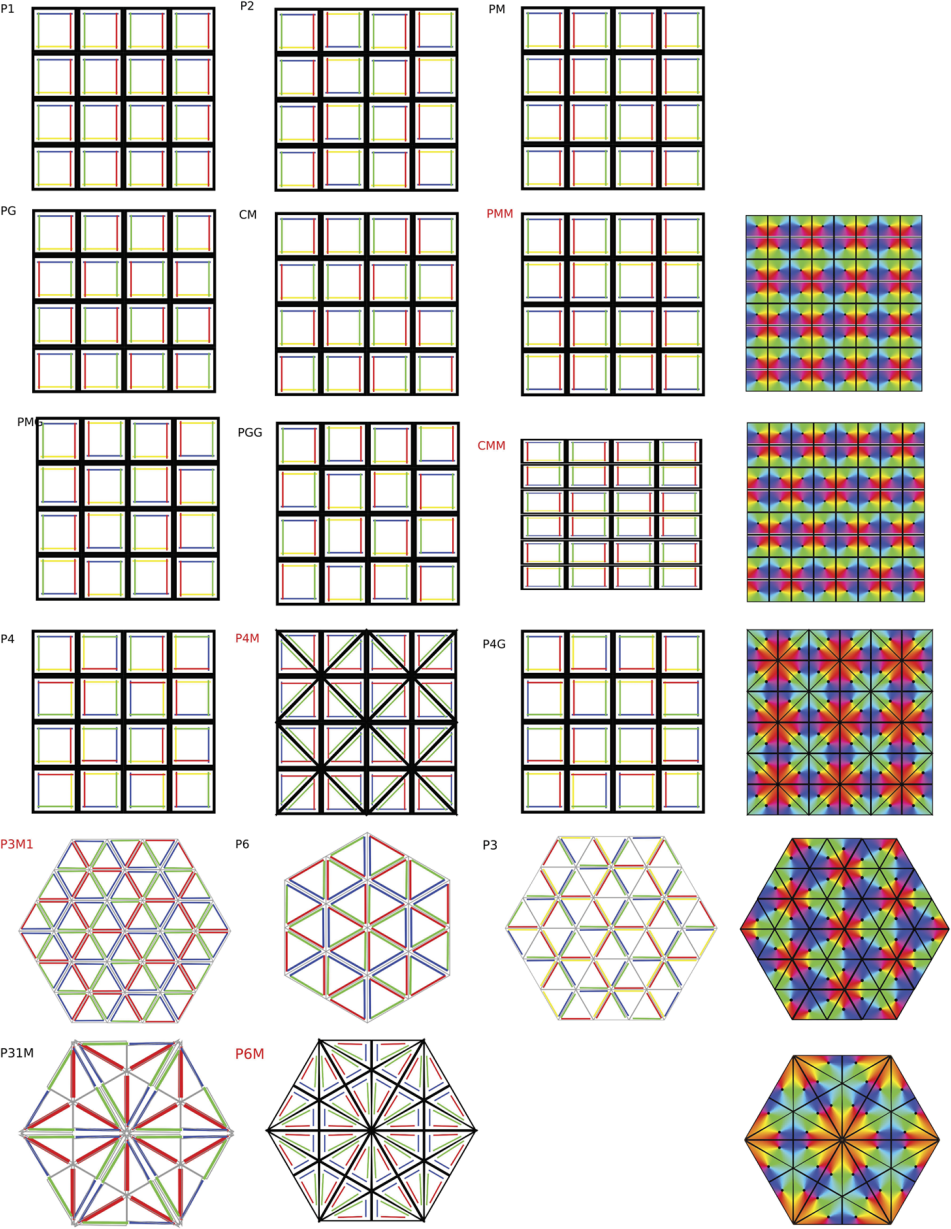
The 17 planar tilings. Each elementary cell represents an hypercolumn with a pinwheel at its center. The *colored edges* represent a coarse preferred orientation domain. The names of the five biologically plausible tilings are highlighted in *red* and a more accurate representation is shown on the *right*. The names of the tilings (and the associated wallpaper groups) are the shortened crystallographic notations; see [27]. pmm, cmm, and p3m1 are those that best agree with the experimental data

There are $(2N+1)^{2}$ copies of $\varOmega_{0}$ in *Ω*. We further assume for convenience that $\ell=2\pi$. The number *N* will be adjusted to match the number of elementary cells produced in *Ω* by the bifurcation of patterns when only local connections are active. The largest group of isometries leaving the lattice spanned by $\varOmega_{0}$ invariant is $\mathbf {D}_{k}\ltimes \mathbb {Z}_{2N}^{2}$ ($k=4$ in the square lattice case and $k=6$ in the hexagonal lattice case). Let us now describe how the pinwheels are distributed in *Ω*. Pinwheels are the singular points of the PO map at which no orientation is preferred. Together with the orientation map they introduce an anisotropy in the lattice, reducing the symmetry group $\mathbf {D}_{k}\ltimes \mathbb {Z}_{2N}^{2}$ of the lattice to a *crystallographic* subgroup (see [24, 25], which is called, in the two-dimensional case, a *wallpaper group*.^3^ See [27] for a description of these groups and for the *shortened crystallographic notation,* which is used in the following. It is well known that there are 17 such groups, up to isomorphism, see Fig. 3 for an illustration of these groups and the corresponding tilings.

However, there are restrictions on the number of possible patterns of pinwheels. First, we only consider square and hexagonal lattices. Second, in order to be biologically plausible the patterns built from the orientation map must be continuous except at the pinwheels. We have determined by inspection that over all possibilities, only those five patterns highlighted in Fig. 3 are compatible with these constraints. Among those five we have selected the two which are shown in Fig. 2, i.e. pmm and p3m1, because they best agree with the experimental data. Pinwheels are the points (black/white dots in the pictures) at which all colors meet. Each color in Fig. 2 defines an orientation, which we identify with an angle between $-\pi/2$ and $\pi/2$. In the case (a) of Fig. 2 the tiling is invariant by reflection across the thick black lines (which also give invariance by rotations of angle *π* around their intersection points). In the case (b) the thick black lines are also axes of reflection. Observe that this gives 3-fold rotational symmetries around their points of intersection. The corresponding group is named p3m1. In the following we shall refer to these two PO maps by the names of the associated wallpaper groups.

### Remark 2

We centered the hypercolumns around the pinwheels and used a uniform preferred orientation density such that the proportion of neurons coding for each orientation is the same for each orientation. This is done in order to avoid any preference of the network for a particular orientation. Note that pinwheels come in pairs called counterclockwise and clockwise: at a counterclockwise (respectively, clockwise) pinwheel, the orientation map shows increasing orientations when moving counterclockwise (respectively, clockwise) around it. Finally notice that the fundamental domain is either centered at a pinwheel (Fig. 2(a)), or it is not (Fig. 2(b)).

To be rigorous the set of orientations should be identified with the projective line $P^{1}(\mathbb {R})$. We can further identify $P^{1}(\mathbb {R})$ with the interval $( -\frac{\pi}{2},\frac{\pi}{2} ]$. Let $\mathcal {P}$ be the set of pinwheels in *Ω*, then the PO map is a map

$$\theta:\varOmega\setminus \mathcal {P}\to\biggl( -\frac{\pi}{2},\frac{\pi }{2} \biggr]. $$

Note that $P^{1}(\mathbb {R})$ is diffeomorphic to the circle $S^{1}\simeq( -{\pi},\pi] \bmod2\pi$ through the map $( -\frac{\pi }{2},\frac{\pi}{2} ]\ni\varphi\mapsto2\varphi$. This allows one to naturally define an action of the circle group $S^{1}$ on $P^{1}(\mathbb {R})$: if $\mathbf{R}_{\phi}$ is the rotation of angle $\phi\in S^{1}$ around the origin, then $\mathbf{R}_{\phi}(\varphi)= \varphi+h(\phi )/2$ where *h* is a homomorphism of $S^{1}$. From this, we can define an action of $S^{1}$ on the map *θ* as follows. Let $\phi_{0}=\pi/2$ in the square case and $\phi_{0}=2\pi/3$ in the hexagonal case and let $\mathbf{R}_{\phi}^{\mathbf{p}}$ be the rotation of angle *ϕ* centered on a pinwheel **p**. Then, with the PO maps of Fig. 2,

4 $$ \theta\bigl(\mathbf{R}_{\phi_{0}}^{\mathbf{p}}\mathbf {x}\bigr) = \theta(\mathbf {x})+\epsilon\frac{\phi_{0}}{2}, $$

where $\epsilon=1$ if the pinwheel is counterclockwise and −1 otherwise.

Similarly, let **K** be the reflection w.r.t. a line passing through a pinwheel and parallel to the vector $\mathbf {e}_{1}$ (horizontal axis). For the PO maps of Fig. 2,

5 $$ \theta(\mathbf {K}\mathbf {x})=-\theta(\mathbf {x}). $$

This defines an action of the reflection **K** on the map *θ*. In order to transfer these relations as (possible) symmetries of the network, we use the action of $\mathbf {D}_{k}\ltimes \mathbb {Z}_{2N}^{2}$ on $\mathrm {L}^{2}(\varOmega, \mathbb {R})$:

$$(\gamma,V)\to\bigl(\mathbf {x}\to\gamma\cdot V (\mathbf {x})=V\bigl(\gamma ^{-1}\mathbf {x}\bigr) \bigr). $$

This allows one to write (4) as $(\mathbf {R}_{\phi _{0}}^{\mathbf{p}})^{-1}\cdot\theta=\theta+\epsilon\frac{\phi_{0}}{2}$.

### Remark 3

Note that we could add an arbitrary value $\theta_{0}\in ( -\frac{\pi}{2},\frac{\pi}{2} ]$ to the orientation map and still obtain an orientation map. Except when $\theta_{0}=0$ or $\pm\pi /2$, this would have the effect of breaking the reflection symmetry of *θ*, because applying the new reflection would not preserve the lattice. In the case of the tree shrew, the cortical coordinates (here assumed to be equal to visual field coordinates) must be such that the zero level of the PO map is parallel to the $x_{1}$-axis, because of the anisotropy of the long-range connections [28]. In this case, the reflection **K** acts indeed on the network. On the other hand, for the macaque, any value of $\theta_{0}$ is relevant because the long-range connections are approximately isotropic [29]. Hence in this case the assumption that **K** acts on the network is somewhat artificial. Nevertheless the presence of a reflection symmetry has strong consequences on the dynamics and we shall subsequently consider both the reflection and the non-reflection cases.

We now draw an easy consequence of Eq. (4), which will play a major role in the following analysis.

### Lemma 3.1

*Let*$\alpha \mathbf {e}_{1}$, $\alpha>0$, *be the vector between two closest pinwheels of different types in the direction*$\mathbf {e}_{1}$. *αis the smallest distance between them*. *Then* (4) *implies that*

6 $$ \mathbf {T}_{\mathbf{t}}\cdot\theta(\mathbf {x})=\theta (\mathbf {x}-\mathbf{t}) = \theta(\mathbf {x})+\phi_{0}, \quad\mathbf{t}=\alpha(\mathbf {e}_{1}+ \mathbf {e}_{2}). $$

### Proof

Let us write $\mathbf{R}_{\phi_{0}}^{\mathbf{p}_{cc}}$ (respectively, $\mathbf{R}_{\phi_{0}}^{\mathbf{p}_{c}}$) for the rotation of angle $\phi_{0}$ around a counterclockwise (respectively, clockwise) pinwheel. According to (4) we have $(\mathbf{R}_{\phi_{0}}^{\mathbf{p}_{cc}})^{-1}\cdot\mathbf{R}_{\phi _{0}}^{\mathbf{p}_{c}}\cdot\theta= \theta+\phi_{0}$. From Lemma A.1$(\mathbf{R}_{\phi_{0}}^{\mathbf{p}_{cc}})^{-1}\mathbf {R}_{\phi_{0}}^{\mathbf{p}_{c}}\mathbf {x}= \mathbf {x}+\mathbf{t}$ with $\mathbf{t}= ((\mathbf{R}_{\phi_{0}}^{\mathbf{o}})^{-1}-\mathit{Id})(-\alpha \mathbf {e}_{1})$. This gives $\mathbf{t}= \alpha(\mathbf {e}_{1}+\mathbf {e}_{2})$ and $\mathbf {T}_{\mathbf{t}}\cdot \theta=\theta+\phi_{0}$. □

We have $\alpha=\pi$ in the square case and $\alpha=2\pi/3$ in the hexagonal case (cf. Fig. 2).

### Model and Symmetries of the Long-Range Connections

In macaques, the anisotropy of horizontal connections follows from the anisotropy of the visual field representation in V1, i.e. it is not correlated to a feature like orientation [29]: the patchiness of the connections is hence isotropic. It seems reasonable to assume that this patchiness comes from the connections between populations of neurons which share similar preferred orientations [28]. Our long-range connectivity model reads

7 $$ J_{\mathrm{LR}}(\mathbf {x},\mathbf {y}) = G_{\sigma_{\theta}} \bigl(\theta(\mathbf {x})- \theta(\mathbf {y})\bigr)J_{0}\bigl(\chi,\mathbf{R}^{\mathbf{o}}_{-2\theta (\mathbf {x})} (\mathbf {x}-\mathbf {y})\bigr), $$

where $J_{0}(\chi, \mathbf {x}) = e^{- [(1-\chi)^{2}x_{1}^{2}+x_{2}^{2} ]/2\sigma_{\mathrm{LR}}^{2}}$. When $\chi=0$ the connectivity is isotropic, while when $\chi=1$ it is “the most” anisotropic. $G_{\sigma _{\theta}}$ is a centered Gaussian^4^ with variance $\sigma_{\theta}\approx35^{\circ}$. It produces inhomogeneous patchy connections.

#### Remark 4

The following model of long-range connections was introduced by Bressloff [12]:

8 $$ J_{\mathrm{LR}}^{\mathrm{Bressloff}}(\mathbf {x},\mathbf {y}) = H \bigl(\theta(\mathbf {x}-\mathbf {y})-P_{0}\bigr)J_{0}\bigl(\chi,\mathbf{R}^{\mathbf{o}}_{2\theta(\mathbf {x})} (\mathbf {x}-\mathbf {y})\bigr), $$

where *H* is the Heaviside step function and $P_{0}$ is some constant. It differs mainly^5^ by the first factor, which enforces homogeneity of the connections. It is not clear to us that this factor in (8) enforces connections between neurons with similar preferred orientations. Establishing which one of the two factors proposed in (7) and (8) best agrees with observations requires one to perform more experiments.

We now turn to the examination of the invariance properties of the long-range connections with respect to the symmetries of the lattice of pinwheels and PO map. It is important to emphasize that the symmetries of the long-range connections *differ* from the symmetry groups pmm or p3m1 of the PO maps as we shall see below. The function $J_{\mathrm{LR}}$ is invariant under a transformation $\gamma\in \mathbf {D}_{k}\ltimes \mathbb {Z}_{2N}^{2}$ if $J_{\mathrm{LR}}(\gamma \mathbf {x},\gamma \mathbf {y})=J_{\mathrm{LR}}(\mathbf {x},\mathbf {y})$ for all **x**, **y**. This implies that the equations are equivariant with respect to the action defined in the previous section for the transformation *γ*. We consider successively the cases of square and hexagonal lattices. However, there are some general features which are shared by both types of networks and we start by stating them.

#### Lemma 3.2

*We have*$J_{\mathrm{LR}}(\mathbf{R}^{\mathbf{p}_{cc}}_{\phi_{0}}\mathbf {x},\mathbf {R}^{\mathbf{p}_{cc}}_{\phi_{0}}\mathbf {y})=J_{\mathrm{LR}}(\mathbf {x},\mathbf {y})$.

#### Proof

From (4), the factor $G_{\sigma_{\theta}} (\theta (\mathbf {x})-\theta(\mathbf {y}) )$ is clearly unaffected by the rotations. Now thanks to (4) and the expression $\mathbf {R}^{\mathbf{p}_{cc}}_{\phi_{0}}\mathbf {x}=\mathbf{R}^{\mathbf{o}}_{\phi _{0}}(\mathbf {x}-\mathbf{p}_{cc})+\mathbf{p}_{cc}$,

$$\begin{aligned} J_{0} \bigl( \chi, \mathbf{R}^{\mathbf{o}}_{-2\theta(\mathbf {R}^{\mathbf{p}_{cc}}_{\phi_{0}}\mathbf {x})}\bigl( \mathbf{R}^{\mathbf {p}_{cc}}_{\phi_{0}}\mathbf {x}-\mathbf{R}^{\mathbf{p}_{cc}}_{\phi_{0}} \mathbf {y}\bigr) \bigr) \stackrel{(4)}{=}& J_{0} \bigl( \chi, \mathbf{R}^{\mathbf{o}}_{-2\theta(\mathbf {x})-\phi_{0}}\bigl(\mathbf{R}^{\mathbf{p}_{cc}}_{\phi_{0}} \mathbf {x}-\mathbf{R}^{\mathbf {p}_{cc}}_{\phi_{0}}\mathbf {y}\bigr) \bigr) \\ =& J_{0} \bigl( \chi, \mathbf{R}^{\mathbf{o}}_{-2\theta(\mathbf {x})-\phi _{0}} \mathbf{R}^{\mathbf{o}}_{\phi_{0}}(\mathbf {x}-\mathbf {y}) \bigr) \\ =&J_{0} \bigl( \chi,\mathbf{R}^{\mathbf{o}}_{-2\theta(\mathbf {x})}( \mathbf {x}-\mathbf {y}) \bigr). \end{aligned}$$

 □

Note that in the square case the above result still holds for $\epsilon =-1$ because $2\phi=\pi$ in this case and $J_{0}$ is an even function.

#### Lemma 3.3

*Let***K***be the reflection across the horizontal axis*. *Assume that* (5) *holds*. *Then*$J_{\mathrm{LR}}(\mathbf {K}\mathbf {x},\mathbf {K}\mathbf {y})=J_{\mathrm{LR}}(\mathbf {x},\mathbf {y})$.

#### Proof

The factor $G_{\sigma_{\theta}} (\theta(\mathbf {x})-\theta(\mathbf {y}) )$ is clearly unaffected by the reflection. Now thanks to (5),

$$\begin{aligned} J_{0} \bigl( \chi, \mathbf{R}^{\mathbf{o}}_{-2\theta(\mathbf {K}\mathbf {x})}\mathbf {K}(\mathbf {x}- \mathbf {y}) \bigr) =& J_{0} \bigl( \chi, \mathbf{R}^{\mathbf {o}}_{2\theta(\mathbf {x})} \mathbf {K}(\mathbf {x}-\mathbf {y}) \bigr) = J_{0} \bigl( \chi, \mathbf {K}\mathbf{R}^{\mathbf{o}}_{-2\theta(\mathbf {x})}(\mathbf {x}-\mathbf {y}) \bigr) \\ =& J_{0} \bigl( \chi,\mathbf{R}^{\mathbf{o}}_{-2\theta (\mathbf {x})}( \mathbf {x}-\mathbf {y}) \bigr). \end{aligned}$$

 □

#### Lemma 3.4

*Let***t***be the vector as in Lemma *3.1. *Let*$\gamma= \mathbf {T}_{\mathbf{t}}^{-1}\mathbf {R}^{\mathbf{p}_{c}}_{\phi_{0}}$. *Then*$J_{\mathrm{LR}}(\gamma \mathbf {x}, \gamma \mathbf {y})=J_{\mathrm {LR}}(\mathbf {x},\mathbf {y})$.

#### Proof

We can rewrite Lemma 3.1 as $\mathbf {T}_{\mathbf{t}}^{-1}\cdot\theta=\theta-\phi_{0}$. Hence, we have $\gamma\cdot \theta= \theta-\frac{\phi_{0}}{2}$. We now look at the effect on the long-range connections. The factor $G_{\sigma_{\theta}} (\theta (\mathbf {x})-\theta(\mathbf {y}) )$ is clearly unaffected by the transformation *γ*. Now, thanks to the previous relation:

$$\begin{aligned} J_{0} \bigl( \chi, \mathbf{R}^{\mathbf{o}}_{-2\theta(\gamma^{-1}\mathbf {x})}\bigl( \gamma^{-1}\mathbf {x}-\gamma^{-1}\mathbf {y}\bigr) \bigr) =& J_{0} \bigl( \chi, \mathbf{R}^{\mathbf{o}}_{-2\theta(\gamma^{-1}\mathbf {x})} \bigl( \bigl(\mathbf{R}^{\mathbf{p}_{c}}_{\phi_{0}}\bigr)^{-1}\mathbf {x}-\bigl( \mathbf{R}^{\mathbf {p}_{c}}_{\phi_{0}}\bigr)^{-1}\mathbf {y}\bigr) \bigr) \\ =& J_{0} \bigl( \chi, \mathbf{R}^{\mathbf{o}}_{-2\theta(\gamma ^{-1}\mathbf {x})} \bigl(\mathbf{R}^{\mathbf{p}_{c}}_{-\phi_{0}}\mathbf {x}-\mathbf{R}^{\mathbf {p}_{c}}_{-\phi_{0}} \mathbf {y}\bigr) \bigr) \\ =& J_{0} \bigl( \chi, \mathbf{R}^{\mathbf{o}}_{-2\theta(\gamma ^{-1}\mathbf {x})} \mathbf{R}^{\mathbf{o}}_{-\phi_{0}}(\mathbf {x}-\mathbf {y}) \bigr) \\ =&J_{0} \bigl( \chi, \mathbf{R}^{\mathbf{o}}_{-2(\theta(\mathbf {x})-\phi _{0}/2)-\phi_{0}}(\mathbf {x}- \mathbf {y}) \bigr) \\ =& J_{0} \bigl( \chi,\mathbf{R}^{\mathbf{o}}_{-2\theta(\mathbf {x})}(\mathbf {x}- \mathbf {y}) \bigr). \end{aligned}$$

Applying *γ* to the previous equation gives the *γ* invariance. □

We will use these lemmas to derive the generators of the symmetry group in the square and hexagonal cases. We will also compute the specific crystallographic group that they generate.

#### The Square Case

We are now in a position to derive the symmetry group of the network equations. Note that the case of the Bressloff connectivity (8) leads to a different symmetry group. We start with the generators.

##### Proposition 3.5

*We write*$\mathbf{R}_{\phi_{0}}^{\mathbf{p}_{c}}$*the rotation of angle*$\frac{\pi}{2}$*centered on a clockwise pinwheel*. *For the* (pmm) *PO map in Fig*. 2(a), *the symmetry group associated with the connectivity* (7) *in the case*$\chi >0$, $\epsilon_{\mathrm{LR}}\neq0$*is*:

1. $\varGamma= \langle \mathbf {K}, \mathbf{R}_{\phi_{0}}^{\mathbf {p}_{c}},\mathbf {T}_{\pi(\mathbf {e}_{1}+\mathbf {e}_{2})} \rangle\simeq(\mathbf {D}_{4}\ltimes(\mathbb{Z}/2N\mathbb{Z})^{2} )$*if*$\theta_{0}\in\frac {\pi}{2}\mathbb{Z}$.
2. $\varGamma= \langle\mathbf{R}_{\phi_{0}}^{\mathbf {p}_{c}},\mathbf {T}_{\pi(\mathbf {e}_{1}+\mathbf {e}_{2})} \rangle\simeq(\mathbf {C}_{4}\ltimes (\mathbb{Z}/2N\mathbb{Z})^{2} )$*otherwise*.

*Finally*, *the subgroup of translation symmetries is the lattice*

$$\mathcal{L} \bigl[ \pi(\mathbf {e}_{1}+\mathbf {e}_{2} ),\pi(\mathbf {e}_{1}-\mathbf {e}_{2} ) \bigr]. $$

##### Proof

This is a direct consequences of the lemmas in the previous section. There is, however, a simplification in the square case, namely the translation $\mathbf {T}_{\pi(\mathbf {e}_{1}+\mathbf {e}_{2})}$ is a symmetry. Indeed, using Lemma 3.1, it is straightforward to show that $\mathbf {J}_{\mathrm{LR}}(\mathbf {T}_{\pi(\mathbf {e}_{1}+\mathbf {e}_{2})}x,\mathbf {T}_{\pi (\mathbf {e}_{1}+\mathbf {e}_{2})}y)=\mathbf {J}_{\mathrm{LR}}(x,y)$. Note that the translation $\mathbf {T}_{\pi (\mathbf {e}_{1}+\mathbf {e}_{2})}$ commutes with $\mathbf {T}_{2\pi \mathbf {e}_{1}}$ and $\mathbf{R}_{\phi _{0}}^{\mathbf{p}_{c}}$. Finally, let us show that $\mathbf {T}_{2\pi \mathbf {e}_{1}}$ is generated by the group elements listed in the lemma. Indeed, $\mathbf {T}_{\pi (\mathbf {e}_{1}-\mathbf {e}_{2})}=(\mathbf{R}_{\phi_{0}}^{\mathbf{p}_{c}})^{-1}\mathbf {T}_{\pi (\mathbf {e}_{1}+\mathbf {e}_{2})}$ and $\mathbf {T}_{2\pi \mathbf {e}_{1}} = \mathbf {T}_{\pi(\mathbf {e}_{1}-\mathbf {e}_{2})}+\mathbf {T}_{\pi(\mathbf {e}_{1}+\mathbf {e}_{2})}$.

To prove that the lattice of translations of the symmetry group is $\mathcal{L} [\frac{\pi}{2}(\mathbf {e}_{1}+\mathbf {e}_{2}),\frac{\pi}{2}(\mathbf {e}_{1}-\mathbf {e}_{2}) ]$, we use the relation $\mathbf {R}^{\mathbf {b}}(\mathbf {R}^{\mathbf {a}})^{-1}\mathbf {x}=\mathbf {x}+ (\mathbf {R}^{\mathbf{o}}-\mathit {Id})(\mathbf {a}-\mathbf {b})$ (see Lemma A.1) which is a translation. Using the rotations with axis $\mathbf {a}=0$ and $\mathbf {b}={\pi} \mathbf {e}_{1}$ allows one to conclude. □

The previous result gives the generators of the group. These groups are very well known as crystallographic groups in the literature (see [24, 25, 27] for an introduction). More precisely, we find that the symmetry group *Γ* of the equations, in the case $\chi,\epsilon_{\mathrm{LR}}\neq0$, is the crystallographic group P4M if $\theta_{0}\in\frac{\pi}{2}\mathbb{Z}$ and P4 otherwise.

It is interesting to note that $\mathbf{D}_{2}$ is the point group associated to the pmm PO map, whereas the point group of the network equations can be $\mathbf{D}_{4}$.

#### The Hexagonal Case

The main difference with the square case is that the clockwise rotation is not a network symmetry because of the anisotropic function $J_{0}$ in (7) when $\chi>0$. Hence, only half of the pinwheels are centers of rotation for the network equations (1), namely the counterclockwise pinwheels. A direct consequence of the lemmas in the previous section is the following.

##### Proposition 3.6

*For the* (p3m1) *PO map in Fig*. 2(b), *the symmetry groupΓassociated with the connectivity* (7) *in the case*$\chi>0$, $\epsilon_{\mathrm{LR}}\neq0$*is*:

1. $\varGamma= \langle \mathbf {K}, \mathbf{R}_{\phi_{0}}^{\mathbf {p}_{cc}},\mathbf {T}_{\frac{2\pi}{3}(e_{1}+e_{2})}^{-1}\mathbf{R}_{\phi _{0}}^{\mathbf{p}_{c}} ,\mathbf {T}_{2\pi e_{1}} \rangle$*if*$\theta_{0}\in \frac{\pi}{2}\mathbb{Z}$.
2. $\varGamma= \langle\mathbf{R}_{\phi_{0}}^{\mathbf {p}_{cc}},\mathbf {T}_{\frac{2\pi}{3}(e_{1}+e_{2})}^{-1}\mathbf{R}_{\phi _{0}}^{\mathbf{p}_{c}} ,\mathbf {T}_{2\pi e_{1}} \rangle$*otherwise*.

*Finally*, *the subgroup of translation symmetries is the lattice*$\mathcal{L} [{2\pi}e_{1},{2\pi}e_{2} ]$.

##### Proof

The only thing left to prove is about the lattice of translations. We can look at (see Lemma A.1) $\mathbf {T}_{\mathbf{t}}^{-1}\mathbf {R}^{\mathbf{p}_{c}}(\mathbf {R}^{\mathbf{p}_{cc}})^{-1}\mathbf {x}=\mathbf {x}+ (\mathbf {R}^{\mathbf{o}}-\mathit{Id})(\mathbf{p}_{cc}-\mathbf{p}_{c})-\mathbf{t}$ which is a translation where $\mathbf{t}\equiv\frac{2\pi }{3}(e_{1}+e_{2})$. When computed for $\mathbf{p}_{cc}-\mathbf{p}_{c}\in\{ \frac{2\pi}{3}e_{1},-\frac{2\pi}{3}e_{2},-\frac{4\pi}{3}e_{1}, \frac {4\pi}{3}e_{2}\}$, we do not find a sub-lattice of translations different from $\mathcal{L} [{2\pi}e_{1},{2\pi}e_{2} ]$. □

As for the square lattice, we can identify the group in the hexagonal case. The symmetry group *Γ* of the equations, in the case $\chi ,\epsilon_{\mathrm{LR}}\neq0$, is the crystallographic group p3m1 if $\theta_{0}\in\frac{\pi}{2}\mathbb{Z}$ and P3 otherwise.

## Forced Symmetry Breaking of Patterns

We now study the perturbation of the torus of translated states from $V_{0}$ when it is an attractor. It is well known that in this case, a torus, flow-invariant manifold persists when the equations are perturbed, as long as the perturbations are small (and smooth) enough [30].

Our aim in this section is to determine how many steady-states do actually persist when the system is perturbed by turning on long-range connections $\epsilon_{\mathrm{LR}}\neq0$, and which phase portrait is observed on the perturbed torus.

Our method is as follows. We first analyze the remaining symmetries on the perturbed torus when $\epsilon_{\mathrm{LR}}\neq0$. This allows one to assert the persistence of some steady-states (equilibria) which stand for points of maximal isotropy for the action of *Γ* on the perturbed torus. Moreover, when these isotropy subgroups contain the *m*-fold rotation group $\mathbf {C}_{m}$ with $m\geq3$, these equilibria are foci (attractive or repulsive) or nodes (the case when the Jacobian matrix has a double real eigenvalue). Now, the topology of the torus is an important constraint for the distribution of equilibria of saddle and other types. This follows from the *Poincaré–Hopf theorem*, which can be found in an abundance of literature and textbooks, and which can be stated as follows:

### Theorem 4.1

*Let*$\mathcal{V}$*be a compact orientable surface and suppose that all equilibria of a vector field defined on*$\mathcal{V}$*are non*-*degenerate* (*i*.*e*. *hyperbolic*: *all eigenvalues of Jacobian matrices have non*-*zero real part*). *Letnbe the number of equilibria on*$\mathcal{V}$*andpthe number of those which are of* saddle *type* (*eigenvalues have opposite signs*). *Then*$n-2p=\chi$, *the Euler characteristic of*$\mathcal{V}$.

In our case $\mathcal{V}$ is the torus, and hence $\chi=0$. Therefore there are an equal number of equilibria of saddle type and of non-saddle type. These two pieces informations (symmetries and Poincaré–Hopf theorem) greatly help to classify the possible phase portraits on the torus. The idea of analyzing the dynamics on a group orbit of equilibria under symmetry-breaking perturbations was introduced by [16]. It was applied in a theoretical setting to the case when the group orbit is a torus of square patterns [31] or hexagonal patterns [32] with the aim of showing the existence of robust heteroclinic cycles under certain conditions. Our aim here is different and we only focus on the cases which correspond to our model.

We next consider the square and hexagonal cases and we list the simplest possible phase portraits, that is, with the minimal number of equilibria, assuming that these are the pictures which will naturally arise in our model, unless further degeneracies are assumed. Then direct numerical simulations of the dynamics on the perturbed torus allow us to fix the actual dynamics which is induced on the invariant tori when switching on the long-range connections. Then direct numerical simulations of the dynamics on the perturbed torus allow us to select the dynamics, among the ones we predicted, which is induced on the invariant tori when switching on the long-range connections.

### On the Perturbed Torus

**Fig. 4:**
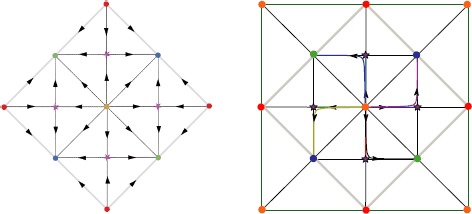
Example of dynamics for the PO map in Fig. 2(a) in the case where $\varGamma= \langle \mathbf {R}_{s},\mathbf {T}_{0,2\pi} \rangle$ and $\theta_{0}=0$. *Left*: Sketch of the phase portrait in the fundamental domain. *Right*: Eight numerical trajectories of (1) projected on the unperturbed torus $\mathcal{T}_{0}$. The fundamental domain is superimposed to compare with prediction. The parameters are as follows: connectivity $J_{\mathrm{LR}}^{p,r}(\mathbf {x},\mathbf {y})$ (see Appendix B), $\epsilon_{\mathrm{LR}}=0.001$, $\chi=0.9$, and $\sigma=1.06264$. The size of the cortex is $4\times2\pi$. The numerical mesh size is 1024^2^

As the perturbed torus $\mathcal{T}_{\epsilon_{\mathrm{LR}}}$ is diffeomorphic to the unperturbed torus $\mathcal{T}_{0}$, we can work in the coordinates of $\mathcal{T}_{0}$ for which the group action expression is known (see below). In the numerical experiments, the diffeomorphism is not known and thus, as we use $\mathcal{T}_{0}$ coordinates, the projection of the patterns on this coordinate system is only approximative (see Fig. 4, for example).

To fix ideas, it is useful to be a bit more explicit. When $\epsilon _{\mathrm{LR}}=0$, we write the unperturbed torus as

$$\mathcal{T}_{0} = \bigl\lbrace V_{0}(\cdot-\mathbf{t}), \mathbf{t}\in\varOmega\bigr\rbrace \subset \mathrm{L}^{2}_{\mathrm {per}}(\varOmega, \mathbb {R}), $$

where $V_{0}$ is a stationary solution for $\epsilon_{\mathrm{LR}}=0$. We assume that the torus solution is invariant by rotation and reflection which is equivalent to assuming $V_{0}$ invariant by rotation and reflection. Under this assumption, the actions of rotations/reflections on the torus satisfy

9 $$ \bigl(\mathbf {R}^{\mathbf{o}},\mathbf{t}\bigr)\to \mathbf {R}^{\mathbf{o}} \cdot\mathbf{t},\quad\quad(\mathbf {K},\mathbf{t})\to \mathbf {K}\cdot\mathbf {t} . $$

This follows from $\mathbf {R}^{\mathbf{o}}\mathbf {T}_{\mathbf{t}}\cdot V_{0} = \mathbf {T}_{\mathbf {R}^{\mathbf{o}}\mathbf{t}}V_{0}$. It implies that the action of a rotation $\mathbf {R}^{\mathbf {a}}$ of axis **a** is given by

$$\bigl(\mathbf {R}^{\mathbf {a}},\mathbf{t}\bigr)\to \mathbf {R}^{\mathbf {a}}\cdot\mathbf{t}. $$

In our model, the lattice of translations symmetries of the torus matches the one of the PO map (see Sect. 2). Hence, $V_{0}$ has the pinwheel periodicity:

10 $$ V_{0}(\mathbf {x}+2\pi k e_{1}+2\pi p e_{2})=V_{0}(\mathbf {x}), \quad\forall \mathbf {x}\in\varOmega, k,p\in\mathbb{Z}. $$

If we identify $V_{0}(\cdot-\mathbf{t})$ and **t**, we can further simplify the study of the perturbed torus by decomposing **t** as follows:

$$\mathbf{t}=\phi_{1} e_{1}+\phi_{2} e_{2}. $$

The assumption (10) implies that $\phi_{i}\in[0,2\pi)$.

#### Remark 5

We cannot apply directly our method to the branch of stripes in Fig. 1, nor to the branch of hexagonal patterns, because the unperturbed torus generated by these patterns is not invariant by rotations.

### Square Case

Using the decomposition $\mathbf{t}= \phi_{1} e_{1}+\phi_{2} e_{2}$, one finds:

11 $$ \begin{cases} \mathbf{R}^{\mathbf{o}}\cdot(\phi_{1},\phi_{2}) =(-\phi_{2},\phi_{1}),\\ \mathbf {K}\cdot(\phi_{1},\phi_{2}) =(\phi_{1},-\phi_{2}). \end{cases} $$

We collect the main results concerning the dynamics on the square in the following proposition. It is the backbone for determining the possible phase portraits on the perturbed torus.

#### Proposition 4.2

*Let us assume that there is a finite number of equilibria on the perturbed torus which are all non*-*degenerate when*$\epsilon_{\mathrm {LR}}\neq 0$, $\chi\geq0$. *For the lattice*pmm, *there are at least eight equilibria on the perturbed torus*$\mathcal{T}_{\epsilon_{\mathrm {LR}}}$, *four of which are saddle*, *and the other four are nodes*/*foci*. *They are given by*

12 $$ \operatorname{Fix}\bigl(\bigl\langle \bigl( \mathbf{R}^{\mathbf{o}} \bigr)^{2}\bigr\rangle \bigr) = \bigl\lbrace (0,0), (0,\pi), (\pi,0), (\pi,\pi) \bigr\rbrace , $$

*which are centers of rotation*.

#### Proof

It is easy to prove (12). Fixed point subspaces are flow invariant, this implies that $\operatorname{Fix}(\langle(\mathbf {R}^{\mathbf{o}} )^{2}\rangle)$ is composed of stationary solutions. We also note that

$$\operatorname{Fix}\bigl(\bigl\langle \mathbf{R}^{\mathbf{o}}\bigr\rangle \bigr)= \bigl\lbrace (0,0), (\pi,\pi) \bigr\rbrace . $$

We write $\frac{d}{dt}{\phi} = F({\phi})$ the dynamics on the torus. The equivariance implies that $dF(\gamma\cdot\phi)\gamma=\gamma\cdot dF(\phi)$. As ${\phi}\in\operatorname{Fix}(\langle\mathbf{R}^{\mathbf{o}}\rangle)$ is *Γ*-invariant, it implies that $dF(\phi)$ commutes with the rotation (11). Simple linear algebra shows that $dF(\phi)$ must be a rotation matrix, i.e. that $\operatorname{Fix}(\langle \mathbf {R}^{\mathbf{o}}\rangle)$ is composed of nodes/foci. It remains to show that this is also true for $(0,\pi)$ and $(\pi,0)$. This follows from Lemma 3.4 and

$$\mathbf {T}_{\pi(e_{1}+e_{2})}^{-1}\mathbf{R}^{\mathbf{o}}\cdot(0,\pi) = (0, \pi). $$

As $\mathbf{R}^{\mathbf{o}}$ and $\mathbf {T}_{\pi(e_{1}+e_{2})}$ commute, $\mathbf {T}_{\pi(e_{1}+e_{2})}^{-1}\mathbf{R}^{\mathbf{o}}$ is of order 4, hence it is the rotation of center $(0,\pi)$. Now, we can see that the action of $\mathbf {T}_{\pi(e_{1}+e_{2})}^{-1}\mathbf{R}^{\mathbf{o}}$ on the manifold $\mathcal{T}_{0}$ is affine. Writing $\gamma\equiv \mathbf {T}_{\pi (e_{1}+e_{2})}^{-1}\mathbf{R}^{\mathbf{o}}$, the equivariance gives

$$d\gamma\bigl(F(0,\pi)\bigr)\,dF(0,\pi) = dF\bigl(\gamma(0,\pi )\bigr)\,d\gamma(0, \pi). $$

From *γ* being affine and $(0,\pi)\in\operatorname{Fix}(\gamma )$, we find that $dF(0,\pi)$ commute with $d\gamma=d\mathbf{R}^{o}$ seen as a map on the torus. This allows one to conclude that $(0,\pi)$ is a node/focus, and also $(\pi,0)$. Being fixed points of rotations, the four nodes/foci are center of rotation symmetry.

Assume now that there are a finite number of zeros $(\phi _{i})_{i=1,\ldots,n}$ on $\mathcal{T}_{\epsilon_{\mathrm{LR}}}$ which are all non-degenerate. Thanks to Theorem 4.1

$$\sum_{i=1}^{n}\operatorname{sign}\det dF({ \phi_{i}})=0. $$

This gives

$$-4 = \sum_{\phi_{i}\notin\operatorname{Fix} (\mathbf {R}_{s}^{2})}\operatorname{sign}\det dF({\phi_{i}}), $$

which implies the existence of at least four saddles. □

A convenient way to find the foci is to look at the fundamental domain in Fig. 2(a). These foci corresponds to the pinwheels in the fundamental domain.

**Fig. 5:**
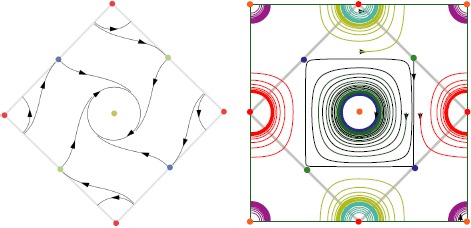
Example of dynamics for the PO map in Fig. 2(a) in the case where $\varGamma= \langle \mathbf {R}_{s},\mathbf {T}_{0,2\pi} \rangle$ and $\theta_{0}\notin\frac{\pi}{2}\mathbb{Z}$. *Left*: Simplest dynamics found in this case shown in the fundamental domain. *Right*: Numerical solution of (1) projected on the unperturbed torus $\mathcal{T}_{0}$. The fundamental domain is superimposed to compare with prediction. The parameters are as follows: connectivity $J_{\mathrm{LR}}^{b,a}(\mathbf {x},\mathbf {y})$ (see Appendix B), $\epsilon_{\mathrm{LR}}=0.001$, $\chi=0.9$, and $\sigma=1.06264$. The size of the cortex is $4\times2\pi$. The numerical mesh size is 1024^2^

We have seen that the minimal configuration, under the hypothesis that all equilibria on the perturbed torus are hyperbolic, is that of eight equilibrium points with four foci and four saddles. How does the associated phase portrait look like on the torus? The answer crucially depends upon the presence of the reflection symmetry (case when $\theta _{0}=0$). In this case the axes of reflection symmetry go through the equilibria. Therefore the foci are necessarily of node type: the eigenvalues of the Jacobian are double and real. The axes of reflection symmetry are invariant by the flow, which constrains the phase portrait to look like the one shown in Fig. 4. On the other hand when there is no reflection symmetry the foci are typically “true” foci: the eigenvalues of the Jacobian are complex conjugate. This allows for the possibility of periodic orbits centered at such foci, as shown in Fig. 5. These two typical situations are observed numerically, as shown on the right pictures in Figs. 4 and 5.

In order to observe limit cycles numerically, we had to change the connectivity. Indeed, if we use the prefactor $G_{\sigma_{\theta}}=\cos $ in (7), the imaginary part of the eigenvalues of the equilibria coming from the breaking of the reflection symmetry by choosing $\theta_{0}\notin\frac{\pi}{2}\mathbb{Z}$ is tiny: at least 3 orders of magnitude smaller than the real part. In effect, even if we break the reflection symmetry, we observe a dynamics similar to the one in Fig. 4. To have larger imaginary parts, we connect neurons with opposite preferred orientation by choosing the prefactor $G_{\sigma_{\theta}}=\sin$ in (7). We further choose the connectivity with largest imaginary part among connectivities in Appendix B. Note that despite varying almost all parameters, we only observed the two situations as in Figs. 4 and 5 (up to a time reversal of the absence of limit cycles), as if naturally, the network equations (1) produced the simplest possibilities for all parameters that we investigated.

#### Remark 6

We would like to mention that great care was taken to code the equivariance and that numerically, the error on symmetries was around 10^−16^ for the 2-norm of an arbitrary vector of 2-norm around 37. The numerical errors on the equivariance relations comes mainly from the PO map *θ* (see Eqs. (4)–(6)). Therefore, we computed the PO map by first building its fundamental domain by rotating/reflecting a basic cell and then padding this fundamental domain to cover *Ω*. This numerical accuracy of the equivariance allows one to check the predicted values of the stationary points with great accuracy using a Newton algorithm. In particular, we find numerically in Fig. 4 that the points $(\mathbf {R}^{\mathbf{o}} )^{k}\cdot(\frac{\pi}{2},0 )$, $k=0,\ldots, 3$ are indeed saddle points.

### Hexagonal Case

We now consider the hexagonal lattice. This case is different from the square lattice because it seems from Proposition 3.6 that only counterclockwise pinwheels are center of rotations. However, it turns out that *γ* as in Lemma 3.4 is a rotation on the torus, hence yielding the other pinwheels as center of rotations.

We prove the next proposition using a different method from the one used in the proof of Proposition 4.2. Using a decomposition $\mathbf{t}= \phi_{1} e_{1}+\phi_{2}e_{2}$, one finds

$$\mathbf {R}^{\mathbf{p}}\cdot\mathbf{t}=\left [ \begin{matrix} 2p_{1}+p_{2}-\phi_{1}-\phi_{2} \\ \phi_{1}-p_{1}+p_{2} \end{matrix} \right ],\quad\mathbf{p} = p_{1} e_{1}+p_{2}e_{2}. $$

#### Proposition 4.3

*Let us assume that there is a finite number of equilibria on the perturbed torus which are all non*-*degenerate when*$\epsilon_{\mathrm{LR}}\neq 0$, $\chi\geq0$. *For the lattice*p3m1, *there are at least* 18 *equilibria on the perturbed torus*$\mathcal{T}_{\epsilon_{\mathrm {LR}}}$, *nine of which are saddle and the other nine are nodes*/*foci*, *given by the lattice*$\mathcal{L} [\frac{2\pi}{3}e_{1},\frac{2\pi }{3}e_{2} ]$*which are centers of rotation*. *The subgroup of translation symmetries is the lattice*$\mathcal{L} [\frac{2\pi }{3} (\mathbf {e}_{1}+\mathbf {e}_{2} ),\frac{2\pi}{3} (-\mathbf {e}_{1}+2\mathbf {e}_{2} ) ]$.

#### Proof

In the fundamental domain, only counterclockwise pinwheels lead to a rotational symmetry. The center of rotation is then a node/focus point. In particular, we find the following node/foci points (see Sect. 3.1.2 for a definition)

$$(\phi_{1},\phi_{2})\in\biggl\{ \biggl( \frac{4\pi}{3},0 \biggr), \biggl(\frac{2\pi}{3},\frac{4\pi}{3} \biggr), \biggl(0,\frac{2\pi }{3} \biggr) \biggr\} . $$

We now look at the symmetry $\gamma\equiv \mathbf {T}_{\frac{2\pi }{3}(e_{1}+e_{2})}^{-1}\mathbf {R}^{\mathbf{p}_{c}}$ defined in Proposition 3.6, where the axis of rotation is $\mathbf{p}_{c} = \frac{2\pi}{3}(2e_{1}+e_{2})$. On the hexagonal torus, we find that $\gamma=\mathbf {R}^{\mathbf{o}}$, which can be seen by writing the equations in the basis $e_{1}$, $e_{2}$. It yields $\operatorname{Fix}(\gamma)= (\frac{\pi }{3},\frac{\pi}{3} )\mathbb{Z}$. Hence, these points are equilibria of node/foci type. It gives three additional nodes/foci.

We can use these centers of rotation to rotate each node/foci in order to find other equilibria. Using Lemma A.2, we have $\gamma=\mathbf {R}^{\mathbf {v}}\cdot \mathbf {R}^{\mathbf{o}}= (\mathbf {R}^{\mathbf{u}} )^{-1}$ where $\mathbf {v}=\frac{2\pi}{3}(e_{1}+2e_{2})$ and $\mathbf{u}=\frac{4\pi}{3}e_{2}$. This yields the additional centers of rotations (hence node/foci):

$$(\phi_{1},\phi_{2})\in\biggl\{ \biggl(\frac{2\pi}{3},0 \biggr), \biggl(0,\frac{4\pi}{3} \biggr), \biggl(\frac{4\pi}{3}, \frac{2\pi }{3} \biggr) \biggr\} . $$

It follows that there are (at least) nine foci. Assuming that there is a finite number of zeros on $\mathcal{T}_{\epsilon_{\mathrm{LR}}}$ which are all non-degenerate, Theorem 4.1 implies that there are as many saddles as foci.

We have shown that $\mathcal{L} [\frac{2\pi}{3}e_{1},\frac{2\pi }{3}e_{2} ]$ is composed of centers of rotation. Using again Lemma A.1 with $\mathbf {a}-\mathbf {b}\in\mathcal{L} [\frac {2\pi}{3}e_{1},\frac{2\pi}{3}e_{2} ]$, we find that the subgroup of translation symmetries is given by $\mathcal{L} [\frac{2\pi }{3} (\mathbf {e}_{1}+\mathbf {e}_{2} ),\frac{2\pi}{3} (-\mathbf {e}_{1}+2\mathbf {e}_{2} ) ]$. □

**Fig. 6:**
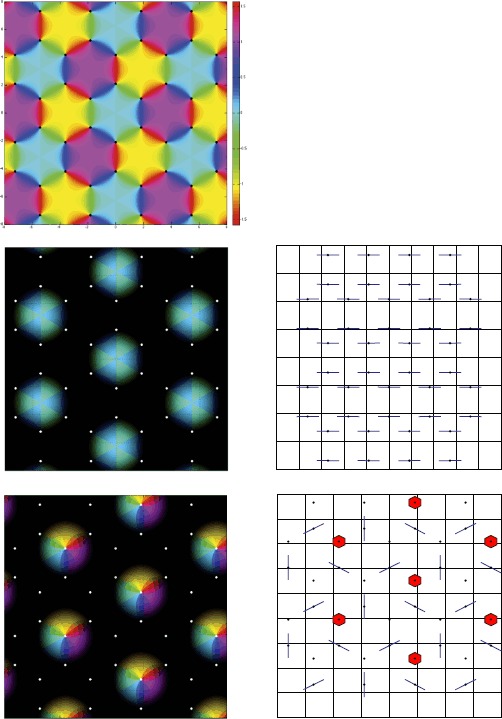
*Top*: The hexagonal p3m1 PO map. *Left column*: two examples of cortical activations, as predicted by Proposition 3.6, overlaid on the PO map. The pinwheels are shown in *white*. *Right column*: interpretation of these activations in cortical coordinates, obtained by pooling the different activated orientations around each pinwheel

We take the opportunity to show how the different nodes/foci found in Proposition 3.6 can be interpreted in cortical coordinates, as shown in Fig. 6. Briefly, we add the cortical activity as a semi-transparent overlay (transparent is when the cortical activity is high) to the PO map *θ*. We then plot small edges or hexagonal patches depending on which a subset of the hypercolumn is activated.

**Fig. 7:**
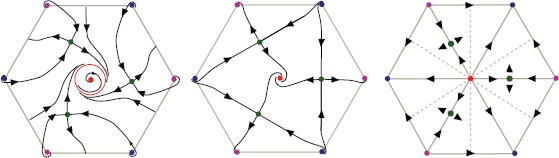
Sketch of the possible phase portraits on the perturbed torus. One case (*left*) is shown, assuming that a periodic orbit surrounds the focus at the center. Another case (*right*) is shown in the case of reflection symmetry. The presence or absence of periodic orbits is essentially related to the sign of the real part of the eigenvalues of the Jacobian at the central focus

As in the square case, we can deduce from these results the possible phase portraits when assuming that the equilibria on the perturbed torus are all hyperbolic and that there are exactly 18 of them, nine foci and nine saddles. The situation is slightly more complicated than in the square case, but it is not difficult to show that a typical phase portrait looks like one of the diagrams shown in Fig. 7. It is numerically checked that this “minimal” situation indeed occurs.

**Fig. 8:**
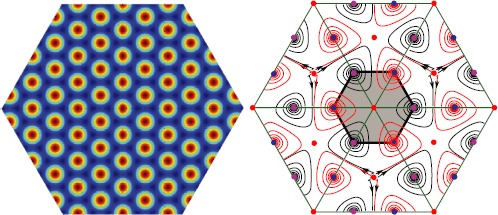
*Left*: Example of solution on the unperturbed torus $\mathcal{T}_{0}$ obtained with a Newton algorithm. *Right*: Numerical solution of (1) projected on the unperturbed torus $\mathcal{T}_{0}$. The *green hexagon* is the fundamental domain from Proposition 4.3. The *bottom right parallelogram* is the result of six simulations, the *hexagon* was built by tiling this *parallelogram*. The parameters are as follows: connectivity $J_{\mathrm{LR}}^{p,a}(\mathbf {x},\mathbf {y})$ (see Appendix B), $\epsilon_{\mathrm{LR}}=0.5$, $\chi=0.2$, and $\sigma=1.11264$. The size of the cortex is $8\times2\pi$. The numerical mesh size is $3\cdot1024^{2}$

As in the square case, we had to use $G_{\sigma_{\theta}}=\sin$ in order to see foci with non-vanishing rotation number. Compared to the square case, we had more difficulty to keep small enough errors in the equivariance relations. This numerical error is 3 orders of magnitude bigger than for the square lattice^6^ and given that we need to numerically solve (1) for a very long time, the errors of the symmetries seem to build up. Nevertheless, we were still able to produce simulations, corresponding to one of the possible diagrams (see Fig. 7). Except for the points $(\frac{2\pi}{3},\frac {2\pi}{3} )\mathbb{Z}$, we find the stationary points predicted by Proposition 3.6 using a Newton algorithm. Around the points $(\frac{2\pi}{3},\frac{2\pi}{3} )\mathbb{Z}$, the Newton algorithm does not converge: this seems to be caused by the presence of saddle points (also predicted in Proposition 3.6 which produces the small kinks on the trajectories around the red points (see Fig. 8)).

In the numerical simulation displayed in Fig. 8, no periodic orbit was found. The simulation seems to correspond to the predicted dynamics shown in Fig. 7, middle. On the other hand, we observe the remarkable fact that the simulations also lead to simplest possible scenarios with the smallest number of stationary points and a periodic solution.

## Discussion

In this work, we have extended the seminal and very influential work [8] on cortical hallucinations after the original work of Ermentrout and Cowan [7]. Our idea is to assume a discrete lattice of pinwheels (as opposed to a continuous one) and to describe the cortical activity in the laminar zone which is located in between pinwheels. This is important, for example, if we want to confront our predictions to optical imaging experiments. This approach has led us to shed some new light onto two outstanding questions. First, what are the possible lattices of pinwheels? They turn out to be closely related to the wallpaper groups. Second, what is the simplest spontaneous dynamics generated by these networks? They turn out to be determined by the perturbation of invariant tori.

The first question is natural but, to the best of our knowledge, has never been addressed theoretically, despite the fact that it allows one to apply the equivariant theory of dynamical systems in a very similar way to [8] albeit in a more biologically plausible setting. In [33] the authors describe a mechanism that allows them to describe the probability of observing a network of pinwheels with a given density, but because their equations are driven by white noise they lose all symmetries.

The second question is more subtle and in effect stems from numerical work where we found it very difficult to implement the ideas of [8, 12], at least for a square cortex. The computation of the bifurcation diagram in Fig. 1 led us to study the perturbation of solutions that were not close to bifurcation points (see the brown lines in Fig. 1), indeed this accounts for the most probable dynamics. As such, the bifurcation diagram shown in Fig. 1 is an indication of the difficulty to apply the theory of cortical hallucinations developed in [8] where it was assumed that such stereotyped cortical patterns could be explained by adjusting the network parameters close to bifurcations. Indeed, in such a setting, the validity of the theory shrinks rapidly with the size of the cortex and, as far as we know, for all practical purposes it is difficult to use it to account for observations. The problem is the same as in the work described in [12], where the author studies the perturbation of a system close to a bifurcation with a spatial forcing close to resonance. Secondary bifurcations might seriously restrict the validity of the approach.

Let us examine the consequences of our investigations onto our current understanding of the functioning of V1.

The first consequence can be drawn from Fig. 1; the hexagonal case is the “only” robust one from a modeling point of view. Indeed, even with the square lattice, the branches which are stable over an extended range of parameters, have a near hexagonal symmetry. Hence, there is a mismatch between the solution approximate symmetry and the network symmetry, which is only resolved in the case of the hexagonal pinwheel lattice.

The second consequence is that, within the class of pinwheel lattices that we consider, those displaying the reflection symmetry are non-generic (see Sect. 3). This induces the presence of foci and (possibly) limit cycles as described in Propositions 4.2 and 4.3. However, the imaginary part of the eigenvalues at the foci is very small for the excitatory long-range connections that are biologically plausible [28, 29] and this makes the observation of limit cycles difficult.^7^ To increase the imaginary part in simulations and observe limit cycles (see Fig. 5), we artificially use a connectivity that connects neurons with orthogonal preferred orientations e.g. $G_{\sigma_{\theta}}=\sin$. This is different from [8] where all bifurcated patterns are stationary. Note that [11] reports time-periodic states in model similar to [8] but with additional symmetry.

**Fig. 9:**
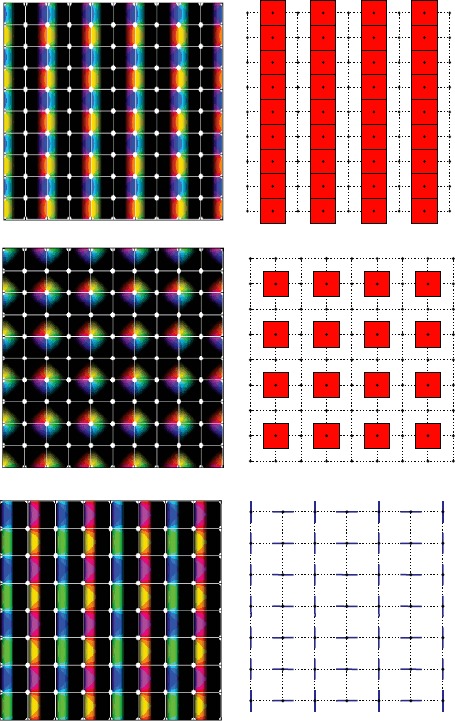
*Left*: Example of cortical activation added as a semi-transparent overlay to a pmm PO map. The pinwheels are shown in white. *Right*: Interpretation of such activation in cortical coordinates made by pooling the different activated orientations around each pinwheel

The last consequence of our work is that it generalizes both theories described in [7] and [8]. Indeed, the first theory allows the description of non-contoured cortical spontaneous activations like in Fig. 9(a)–(b). The second theory generalizes the first one by allowing the prediction of contoured patterns such as those in Fig. 9(c) as well as the non-contoured ones. Our theory describes contoured and non-contoured patterns as well as a mixture of such activation patterns as depicted in Fig. 6(c).

Our work can be extended in several ways. First, there is a need for a bifurcation study of the dynamics on the torus w.r.t. the parameters $\epsilon_{\mathrm{LR}}$, *χ*, etc. Indeed, we have not been very quantitative concerning the role of these parameters in shaping the dynamics. There are also lattices that have not been investigated (cmm, p4m and p6m in Fig. 2) and it would be interesting to study the kind of planforms they can produce. We have not looked at the case where the stripes are stable in the $\mathbf{D}_{4}$-pitchfork. That would be a minor modification of the present work but it would show another type of planforms. More generally, we have not discussed the cases where the unperturbed solution is a circle rather than a torus. Hence, it is desirable to classify the different planforms that can be produced from the unperturbed invariant manifolds for the different lattices. Another extension concerns the study of cases where the lattice of symmetry of the unperturbed torus $\mathcal{T}_{0}$ and the pinwheel lattice differ. The perturbation from the long-range connections would act as a periodic forcing on the unperturbed torus. Some models are available [12, 13], but we believe that they lack an important component: synaptic plasticity. A relatively simple extension would be to consider the effect of synaptic/propagation delays [34, 35]. Synaptic delays [36] will not affect the unperturbed torus but are likely to increase the imaginary part of the eigenvalues which we found to be nonzero albeit small when the reflection symmetry is broken. Other very exciting extensions concern the modeling of the spatial frequency tuning and the ocular dominance domains. It would be very interesting to re-visit some recent work on binocular rivalry [37, 38] in the light of the conclusions presented in this paper.

## Appendix A: Useful Results

### Lemma A.1

Composition of rotations

*Let*$\mathbf {R}^{\mathbf {a}}$ (*respectively*, $\mathbf {R}^{\mathbf {b}}$) *be the rotation of axis***a** (*respectively*, **b**). *Let*$\mathbf {R}^{\mathbf{o}} $*be the rotation by the same angle through the origin*. *Then*

$${\mathbf {R}^{\mathbf {b}}\bigl(\mathbf {R}^{\mathbf {a}}\bigr)^{-1}\mathbf {x}=\mathbf {x}+ \bigl( \mathbf {R}^{\mathbf{o}}-\mathit{Id}\bigr) (\mathbf {a}-\mathbf {b}).} $$

### Proof

Straightforward using $\mathbf {R}^{\mathbf {a}}(\mathbf {x})=\mathbf {R}^{\mathbf{o}}(\mathbf {x}-\mathbf {a})+\mathbf {a}$ and $(\mathbf {R}^{\mathbf {a}})^{-1}(\mathbf {x})=(\mathbf {R}^{\mathbf {o}})^{-1}(\mathbf {x}-\mathbf {a})+\mathbf {a}$. □

### Lemma A.2

*Let*$\mathbf {R}_{h}^{\mathbf {v}}$*the hexagonal rotation of angle*$\frac{2\pi}{3}$*and axis***v***acting on the hexagonal torus*. *Then we have*

$$\mathbf {R}_{h}^{\mathbf {v}}\cdot \mathbf {R}_{h}^{\mathbf{o}}= \bigl( \mathbf {R}_{h}^{\mathbf{u}} \bigr)^{-1},\quad\mathbf{u}=(v_{1}+v_{2},-v_{1}). $$

### Proof

This is elementary algebra when writing the expression of the rotation in the basis $(\mathbf {e}_{1},\mathbf {e}_{2})$. □

## Appendix B: Numerical Experiments

The bifurcation diagram shown in Fig. 1 was computed with the library Trilinos [40] using the FFTw library to compute the FFT on the square lattice. The method implements a Newton–Krylov solver with GMRES linear solver, the eigensolver is based on the Arnoldi algorithm. For the simulation of the full network, we follow [12] to make the connectivity amenable to fast computations, e.g. using FFTs. Indeed, neglecting $G_{\sigma_{\theta}}$ in (7), we find

13 $$ J_{\mathrm{LR}}(\mathbf {x},\mathbf {y}) = \sum_{k=1}^{3}D_{k}( \mathbf {x})J_{k}(\mathbf {x}-\mathbf {y})J(\mathbf {x}-\mathbf {y}), $$

where the mask $J(\mathbf {x}) = \mathbf{1}(\Vert \mathbf {x}\Vert <3\cdot2\pi )-\mathbf{1}(\Vert \mathbf {x}\Vert >1\cdot2\pi)$ allows one to select long-range connections but no local connections. We use two different schemes, the one from [12] (modified by multiplying the PO map *θ* by −1 to induce co-circular preferred connections) and another one found by Taylor expanding $J_{0}$ in (7):

14 $$\begin{aligned} D_{1}^{(b)}(\mathbf {x}) =&\chi\cos^{2}\theta(\mathbf {x}),\quad\quad D_{2}^{(b)}(\mathbf {x})=2\chi\cos\theta(\mathbf {x})\sin\theta(\mathbf {x}), \\ D_{3}^{(b)}(\mathbf {x}) =&\chi\sin^{2}\theta(\mathbf {x}), \quad\quad J_{1}^{(b)}(\mathbf {x})=x_{1}^{2}J(\mathbf {x}), \end{aligned}$$

15 $$\begin{aligned} J_{2}^{(b)}(\mathbf {x}) =&x_{1}x_{2}J(\mathbf {x}),\quad\quad J_{3}^{(b)}(\mathbf {x})=x_{2}^{2}J(\mathbf {x}), \\ D_{1}^{(p)}(\mathbf {x}) =&\cos^{2}\theta(\mathbf {x}),\quad\quad D_{2}^{(p)}(\mathbf {x})=-2\cos\theta(\mathbf {x})\sin\theta(\mathbf {x}), \\ D_{3}^{(p)}(\mathbf {x}) =&\sin^{2}\theta(\mathbf {x}), \quad\quad J_{1}^{(p)}(\mathbf {x})=(1-\chi)x_{1}^{2}+x_{2}^{2}, \\ J_{2}^{(p)}(\mathbf {x}) =&x_{1}x_{2},\quad\quad J_{3}^{(p)}(\mathbf {x})=x_{1}^{2}+(1- \chi)x_{2}^{2}. \end{aligned}$$

In order to take into account the angular selection in $G_{\sigma _{\theta}}(\theta(\mathbf {x})-\theta(\mathbf {y}))$, which is costly to compute, we use the first Fourier component of $G_{\sigma_{\theta}}$, namely

$$J_{\sigma_{\theta}}^{(\mathrm{regular})}(\mathbf {x},\mathbf {y}) \equiv\cos\bigl(\theta(\mathbf {x})-\theta( \mathbf {y})\bigr)=\cos\bigl(\theta(\mathbf {x})\bigr)\cos\bigl(\theta(\mathbf {y})\bigr)+\sin\bigl(\theta (\mathbf {x})\bigr)\sin\bigl(\theta(\mathbf {y})\bigr) $$

or

$$J_{\sigma_{\theta}}^{(\mathrm{anti})}(\mathbf {x},\mathbf {y}) \equiv\sin\bigl(\theta(\mathbf {x})-\theta( \mathbf {y})\bigr)=\sin\bigl(\theta(\mathbf {x})\bigr)\cos\bigl(\theta(\mathbf {y})\bigr)-\cos\bigl(\theta (\mathbf {x})\bigr)\sin\bigl(\theta(\mathbf {y})\bigr). $$

We note that $J_{\sigma_{\theta}}^{(\mathrm{regular})}$ connects neurons with similar preferred orientation, whereas $J_{\sigma_{\theta}}^{(\mathrm{anti})}$ connects neurons whose preferred orientations differ by ±*π*. The reason why we introduce this last connectivity scheme is because it allows one to have eigenvalues of the Jacobian at fixed points with relatively large imaginary parts, which speeds up the dynamics on the invariant torus. To sum up, we use the connectivity function

$$J_{\mathrm{LR}}^{\mathrm{num}}(\mathbf {x},\mathbf {y})=J_{\sigma_{\theta}}(\mathbf {x}, \mathbf {y})J_{\mathrm{LR}}(\mathbf {x},\mathbf {y}), $$

where $J_{\mathrm{LR}}$ is given by (13). It is found that this function satisfies

$$J_{\mathrm{LR}}^{\mathrm{num}}(\mathbf {x},\mathbf {y})=\sum_{k=1}^{6} \tilde{D}_{k}(\mathbf {x}) \tilde{J}_{k}(\mathbf {x}-\mathbf {y})C_{k}(\mathbf {y}), $$

which is computationally very efficient. In the simulations, we use the following connectivities:

16 $$\begin{aligned} J_{\mathrm{LR}}^{b,r}(\mathbf {x},\mathbf {y}) \equiv& J_{\sigma_{\theta}}^{(\mathrm{regular})}(\mathbf {x},\mathbf {y})\sum_{k=1}^{3}D_{k}^{(b)}( \mathbf {x})J_{k}^{(b)}(\mathbf {x}-\mathbf {y})J^{(b)}(\mathbf {x}-\mathbf {y}), \\ J_{\mathrm{LR}}^{b,a}(\mathbf {x},\mathbf {y}) \equiv& J_{\sigma_{\theta}}^{(\mathrm{anti})}( \mathbf {x},\mathbf {y})\sum_{k=1}^{3}D_{k}^{(b)}( \mathbf {x})J_{k}^{(b)}(\mathbf {x}-\mathbf {y})J^{(b)}(\mathbf {x}-\mathbf {y}), \\ J_{\mathrm{LR}}^{p,a}(\mathbf {x},\mathbf {y}) \equiv& J_{\sigma_{\theta}}^{(\mathrm{anti})}( \mathbf {x},\mathbf {y})\sum_{k=1}^{3}D_{k}^{(p)}( \mathbf {x})J_{k}^{(p)}(\mathbf {x}-\mathbf {y})J^{(p)}(\mathbf {x}-\mathbf {y}). \end{aligned}$$

In order to solve the differential equations (1) with a large number of unknowns, we use PETSc [41] and its Python extension PETSc4py [42]. The linear solver in this case is a deflated-GMRES combined with the BDF (backward differentiation formulas) method suitable for stiff systems. The simulations for the square lattice have 1024^2^ unknowns, those for the hexagonal lattice have $3\cdot1024^{2}$.

## Footnotes

1 In effect, doubly periodic solutions.

2 They satisfy our assumption.

3 In our case we could use the equivalent notion of *color group* [26].

4 It is periodic on $( -\frac{\pi}{2},\frac{\pi}{2} ]$; see Appendix B.

5 Note also the difference with (7) in the rotation term. The main reason for the − sign in this term in (7) is to implement co-circularity [20].

6 It is in the order of 10^−13^.

7 Note that we did not study systematically the different biologically plausible connectivities.
